# Nonenzymatic Spontaneous Oxidative Transformation of 5,6-Dihydroxyindole

**DOI:** 10.3390/ijms21197321

**Published:** 2020-10-03

**Authors:** Manickam Sugumaran, Jason Evans, Shosuke Ito, Kazumasa Wakamatsu

**Affiliations:** 1 Department of Biology, University of Massachusetts, Boston, MA 02125, USA; 2 Department of Chemistry, University of Massachusetts, Boston, MA 02125, USA; Jason.evans@umb.edu; 3 Department of Chemistry, Fujita Health University School of Medical Sciences, Toyoake 470-1192, Japan; sito@fujita-hu.ac.jp (S.I.); kwaka@fujita-hu.ac.jp (K.W.)

**Keywords:** melanogenesis, 5,6-dihydroxyindole, oxidative polymerization, DHI melanin, innate immunity, wound healing in arthropods.

## Abstract

Melanin is an important phenolic skin pigment found throughout the animal kingdom. Tyrosine and its hydroxylated product dopa provide the starting material for melanin biosynthesis in all animals. Through a set of well-established reactions, they are converted to 5,6-dihydroxyindole (DHI) and DHI-2-carboxylic acid (DHICA). Oxidative polymerization of these two indoles produces the brown to black eumelanin pigment. The steps associated with these transformations are complicated by the extreme instability of the starting materials and the transient and highly reactive nature of the intermediates. We have used mass spectral studies to explore the nonenzymatic mechanism of oxidative transformation of DHI in water. Our results indicate the facile production of not only dimeric and trimeric products but also higher oligomeric forms of DHI upon exposure to air in solution, even under nonenzymatic conditions. Such instantaneous polymerization of DHI avoids toxicity to self-matter and ensures the much-needed deposition of melanin at (a) the wound site and (b) the infection site in arthropods. The rapid deposition of DHI melanin is advantageous for arthropods given their open circulatory system; the process limits blood loss during wounding and prevents the spread of parasites by encapsulating them in melanin, limiting the damage.

## 1. Introduction

**Figure 1:**
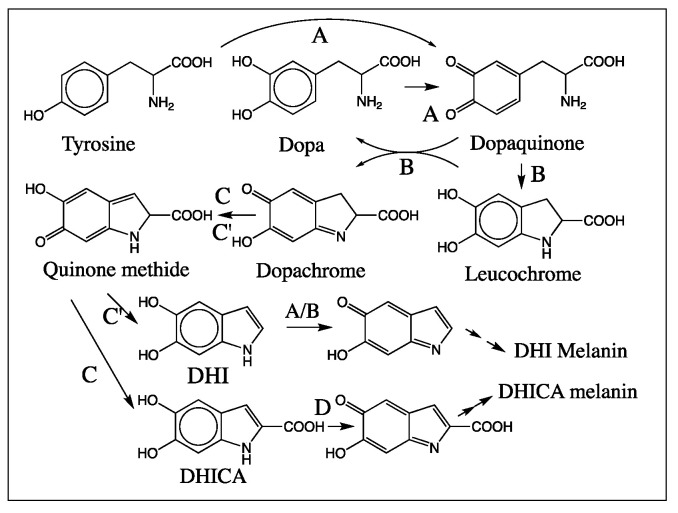
Simplified mechanism for eumelanin biosynthesis. Tyrosinase (**A**) oxidizes both tyrosine and dopa to dopaquinone. Dopaquinone undergoes a nonenzymatic intramolecular cyclization reaction to form leucochrome, which, following redox exchange with dopaquinone itself, produces dopachrome and dopa. Insect dopachrome decarboxylase/tautomerase (**C’**) converts dopachrome to DHI. Mammalian dopachrome tautomerase (**C**) converts dopachrome to DHICA. In mammals, DHI seems to be produced nonenzymatically; in insects, DHICA is apparently not being formed. Oxidative polymerization of DHI and DHICA generates eumelanin pigment. (**B**) represents nonenzymatic reactions; (**D**) represents DHICA oxidase.

The brown to black pigment widely distributed in the skin, hair, and fur of animals is due to the phenolic pigment eumelanin [1,2,3,4,5,6,7,8,9]. It is also ubiquitously present in insects as an exoskeletal pigment and, more importantly, associated with two physiologically important biochemical processes, viz. defense reaction (innate immunity) and wound healing. This makes eumelanin an essential component of an insect’s biochemical arsenal [10,11,12,13,14,15,16,17]. Elaborate studies conducted on the biosynthesis of eumelanin pigment have led to the delineation of its pathway, which is depicted in Figure 1 [1,2,3,4,5,6,7,8,9]. Eumelanin is derived from the amino acid tyrosine and its hydroxylated product dopa. Tyrosinase catalyzes the oxidation of these two compounds and produces dopaquinone, which undergoes rapid intramolecular cyclization and, in turn, generates leucodopachrome. Redox cycling of leucodopachrome by dopaquinone produces a reddish-orange colored dopachrome along with the regeneration of dopa. Although dopachrome was initially believed to undergo a nonenzymatic transformation to 5,6-dihydroxyindole (DHI), studies carried out in mammals and insects revealed the presence of two distinctly different enzymes associated with dopachrome conversion. In mammalian systems, an enzyme catalyzing the conversion of dopachrome to 5,6-dihydroxyindole-2-carboxylic acid (DHICA) was identified and named dopachrome tautomerase [18,19,20,21,22]. For insects, a sister enzyme catalyzing the conversion of dopachrome to DHI was characterized [23,24,25]. Though the insect enzyme catalyzed the decarboxylation of dopachrome, since it also catalyzed the isomerization of a number of dopachrome derivatives, it was named dopachrome decarboxylase/tautomerase [9,25]. Oxidative polymerization of both DHI and DHICA generates the eumelanin pigment (Figure 1).

The initial stage of the eumelanin biosynthetic pathway seems to be well-established. However, delineating the subsequent transformations of DHI and DHICA has proved to be very difficult. Considerable progress has been made due to the pioneering work of Italian scientists. Using matrix-assisted laser desorption/ionization mass spectrometry, these scientists examined the oxidative condensation of DHI and DHICA [26,27,28]. Chemical analysis of the peroxidase/DHI/H_2_O_2_ system revealed the production of dimeric and trimeric adducts of DHI [26,28,29]. Napolitano et al. [28] observed the facile production of dimeric and other oligomeric products during tyrosinase oxidation of DHI. Subsequently, Bertazzo et al. [30,31] reported the presence of different oligomeric products of DHI in the reaction mixture containing tyrosine and tyrosinase. The structure of some of these adducts has been painstakingly solved by these scientists. The dimerization reaction occurs primarily by coupling through 2,4′ and 2,7′ bonding [29,32]. However, the coupling mechanism that produces trimeric and other oligomeric products remains undetermined.

**Figure 2:**
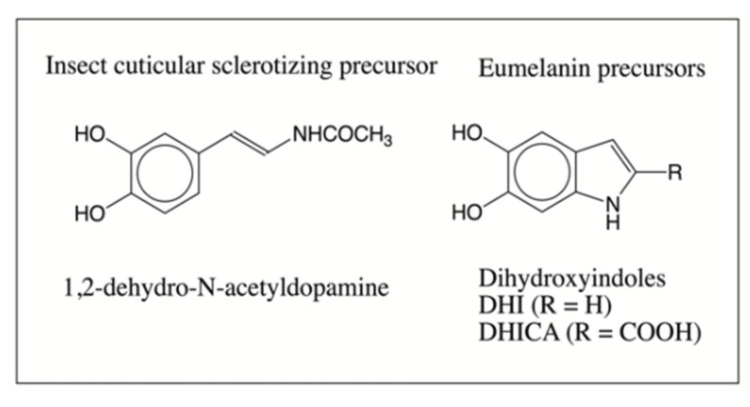
Structure of insect cuticular sclerotizing precursor 1,2-dehydro-*N*-acetyldopamine and eumelanin precursors DHI and DHICA.

In this context, it is important to draw particular attention to two derivatives of dopamine that exhibit similar reactivity. *N*-Acetyldopamine and *N*-β-alanyldopamine are two of the most important catecholamines derivatives that are produced by most, if not all, insects. They are essential components of cuticular sclerotization, which protects all soft-bodied insects against infection and desiccation [15,33]. Sugumaran [14,34] has noted the remarkable similarity between eumelanogenesis and the insect cuticular sclerotization process. For example, both processes are initiated by tyrosinase and related phenoloxidases. Both processes also produce side chain desaturated compounds—dopa gives rise to DHI and DHICA and *N*-acyldopamines generate 1,2-dehydro-*N*-acyldopamine derivatives. Both use similar molecular transformations. The final dihydroxy products formed in both processes exhibit rapid nonenzymatic oxidative polymerization, producing adducts and other oligomers [14,34]. Extensive studies conducted on 1,2-dehydro-*N*-acetyldopamine (deNADA) indicate that this compound, which resembles DHI by having a side chain unsaturation (Figure 2), is highly unstable and readily polymerizes nonenzymatically, even under mild alkaline conditions [35,36,37]. The nonenzymatic aerial oxidation of deNADA leads to dimeric and other oligomeric products via a free radical coupling mechanism. We envisaged that a similar reaction might occur with DHI as well. Surprisingly, such a reaction has not been studied thus far. To fill this void, we examined the initial steps associated with the nonenzymatic oxidative polymerization of DHI. Conventional spectroscopic techniques did not furnish much information about the course of reaction taken by DHI. In the case of deNADA, we were able to obtain a clear picture on the course of oxidative polymerization using mass spectrometric studies [36,37]. Therefore, we decided to examine the nonenzymatic oxidative transformation of DHI using this technique and report the results in this paper.

## 2. Results

### 2.1. Stability of DHI

**Figure 3:**
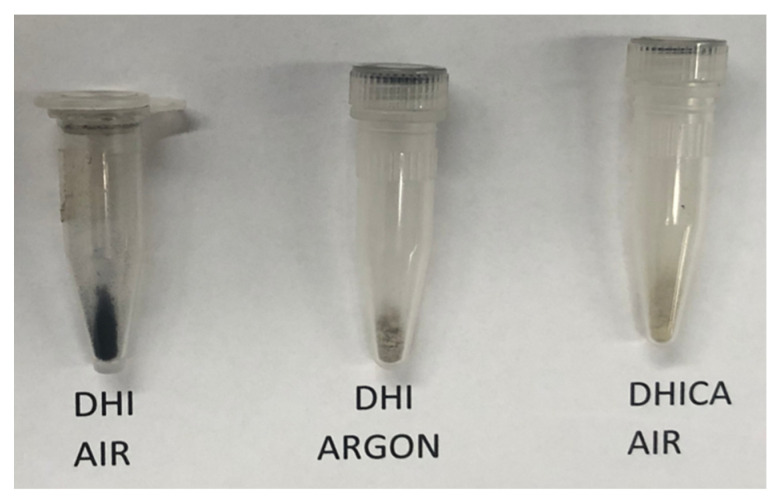
Stability of DHI stored at room temperature for seven days in solid state.

**Figure 4:**
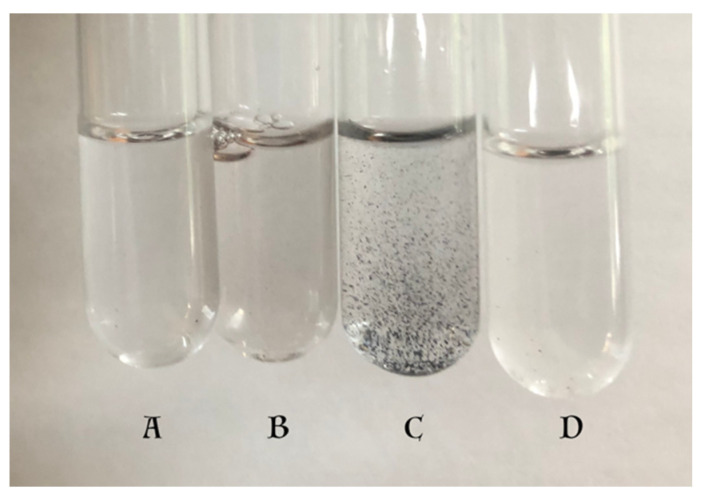
Stability of DHI stored in water at room temperature. (**A**) DHI solution at zero-time. (**B**) DHI solution after 2 h. (**C**) DHI after overnight incubation. (**D**) DHICA stored for 24 h. Note the discoloration of DHI in test tube (**B**). The solution of DHI left in air for 24 h, test tube (**C**), resulted in total precipitation of black-colored eumelanin pigment.

The labile nature of DHI has been well documented [1,7]. DHI prepared and stored under an atmosphere of argon and in the total absence of oxygen stays without polymerization for several months. However, when exposed to air, even in solid state, it suffers facile aerial oxidation and turns into black eumelanin pigment in a matter of days. This discoloration, which is caused by the apparent oxidative polymerization of DHI, occurs even when DHI is stored at −20 °C for 2 months in the atmosphere of air. To prevent such discoloration and polymerization, DHI is routinely stored under an atmosphere of argon. Figure 3 shows the stability of DHI at room temperature. The polymerization of DHI occurs much more rapidly at room temperature; within seven days, DHI becomes dark-colored melanin in the presence of air. Stored under argon, it is much more stable. For comparison, DHICA stored in air is also shown in Figure 3. Note the resistance of DHICA to aerial oxidation and discoloration. Thus, DHI seems to be extremely unstable in comparison with DHICA. Oxidative polymerization of DHI occurs even faster in solution. At neutral pH, DHI polymerizes rapidly, resulting in a darker-colored reaction in 2 h (Figure 4B). Often, such solutions become very clear following the precipitation of black-colored eumelanin pigment when left overnight (Figure 4C). DHICA appeared to be comparatively more resistant to such oxidation and remained colorless for 24 h (Figure 4D).

These preliminary results indicated that DHI is very unstable compared with its carboxylated derivative, DHICA. In order to examine the molecular changes occurring during the exposure of DHI to air, we conducted mass spectrometry of the reaction.

### 2.2. Mass Spectral Studies of DHI Reaction

**Figure 5:**
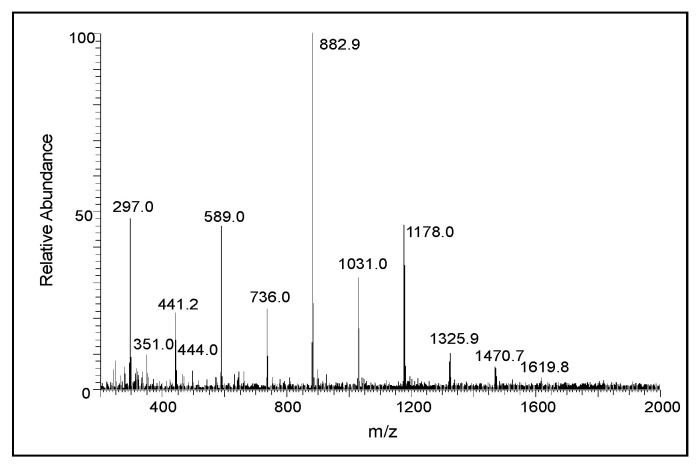
The average electrospray mass spectrum of the DHI reaction after 10 min of incubation at room temperature. Note the presence of clusters of ions corresponding to multiple oligomeric products at regular intervals of about 147 amu.

**Figure 6:**
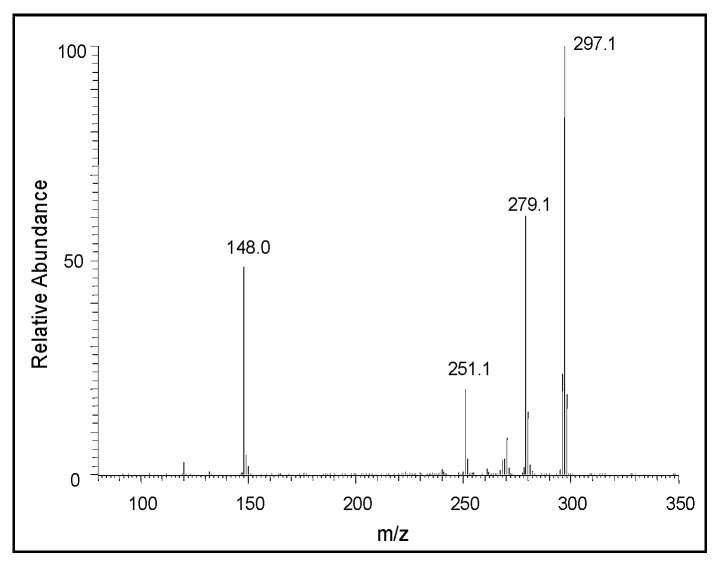
The CID mass spectrum of the ion with an *m/z* value of 297 amu.

Figure 5 shows the average electrospray mass spectra of the DHI reaction. The ion observed at *m/z* 297 is due to the protonated dimeric of DHI, and the ion observed at *m/z* 444 is that of the protonated trimeric product. The next major ion, observed at *m/z* 589, is due to the oxidized form of the protonated tetrameric product. Similarly, the ion present at *m/z* 736 is due to the oxidized form of the protonated pentameric product, and the major ion present at *m/z* 883 is the oxidized form of the protonated hexameric product. The ions observed at *m/z* 1031, 1178, 1325.9, 1470.7, and 1619.8 correspond to the heptameric, octameric, nonameric, decameric, and undecameric products, respectively. To further prove that these ions are due to oligomeric products of DHI, we obtained the Collision-induced decomposition (CID) spectra of the individual ions. Figure 6 shows the CID spectrum of the ion observed at *m/z* 297. The product ion observed at *m/z* 148 is due to the oxidized form of DHI plus a proton. From the fragmentation pattern, it is difficult to draw any conclusions about the actual structure of the adduct(s), as the coupling of the oxidized form of DHI to DHI could occur in different ways, producing multiple isomeric products [26,27].

**Figure 7:**
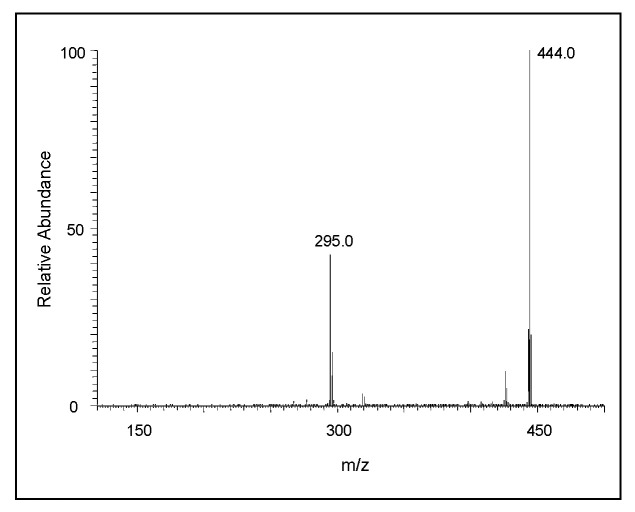
The CID mass spectrum of the ion with an *m/z* value of 444 amu.

**Figure 8:**
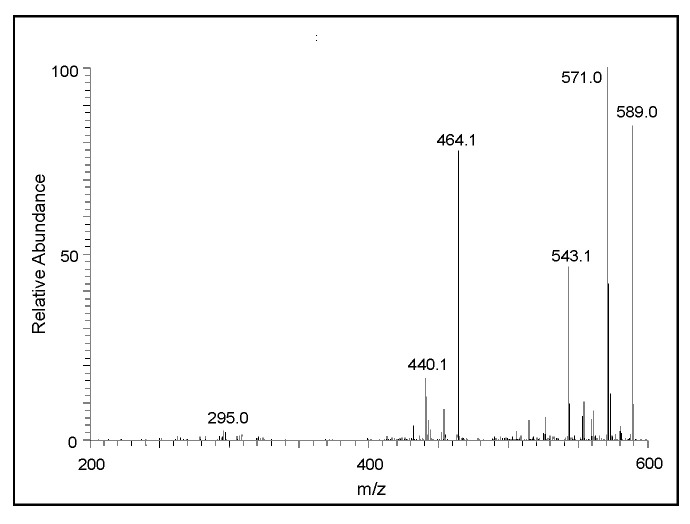
The CID mass spectrum of the ion with an *m/z* value of 589 amu.

The CID spectrum of the *m/z* 444 ion is presented in Figure 7. Its fragmentation pattern yielded a major ion at *m/z* 295 corresponding to the protonated form of the dimeric ion in its two-electron oxidation state. Thus, it can be concluded that the *m/z* 444 ion is due to the trimeric product of DHI. Again, in this case the exact structure of the adduct cannot be deduced from the mass spectral data alone. The CID spectral of the tetrameric product in its oxidized form (*m/z* 589) is shown in Figure 8. One can witness the presence of fragment ions at around *m/z* 440, apparently the oxidized form of the trimeric product, and *m/z* 295, the oxidized form of the dimeric product ion. The loss of 125 amu from 589 seems to produce the ion at *m/z* 464, which could be due to the loss of 3,4-dihydroxyphenylamine group from the M + 1 ion. These overall fragmentation patterns indicate that the parent ion observed at *m/z* 589 is due to the tetrameric form of DHI in its oxidized state.

**Figure 9:**
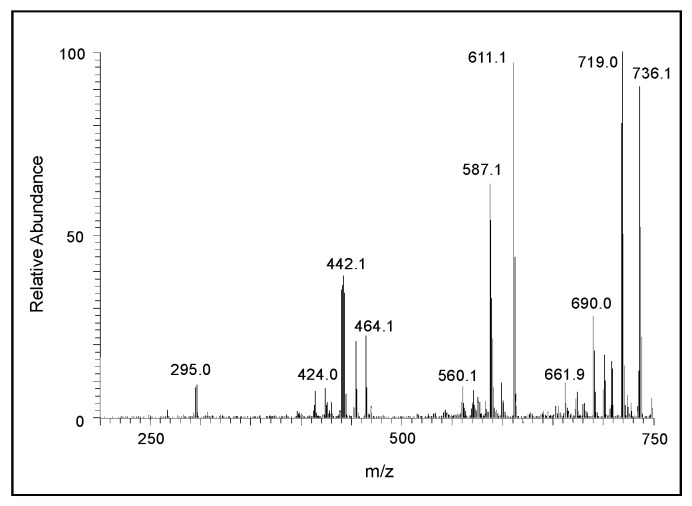
The CID mass spectrum of the ion with an *m/z* value of 736 amu.

**Figure 10:**
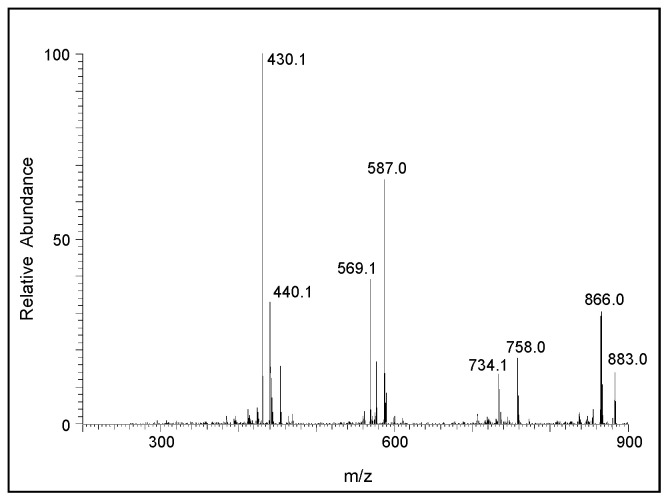
The CID mass spectrum of the ion with an *m/z* value of 883 amu.

**Figure 11:**
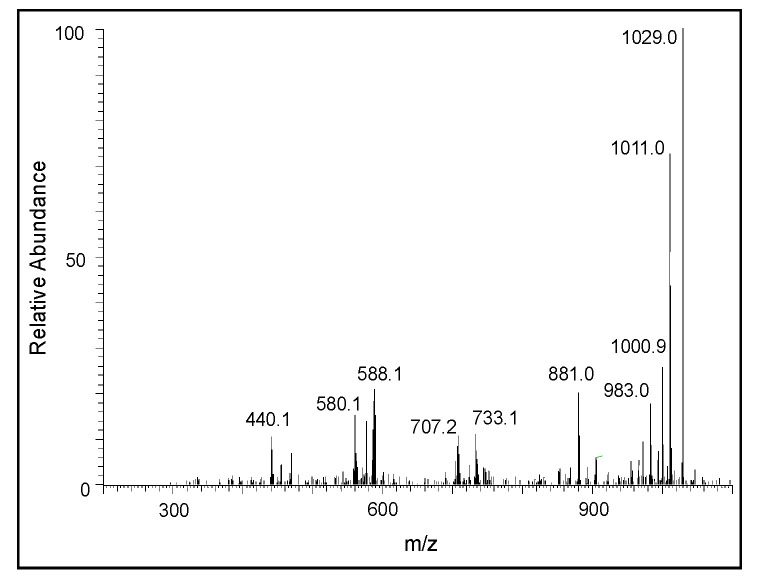
The CID mass spectrum of the ion with an *m/z* value of 1029 amu.

**Figure 12:**
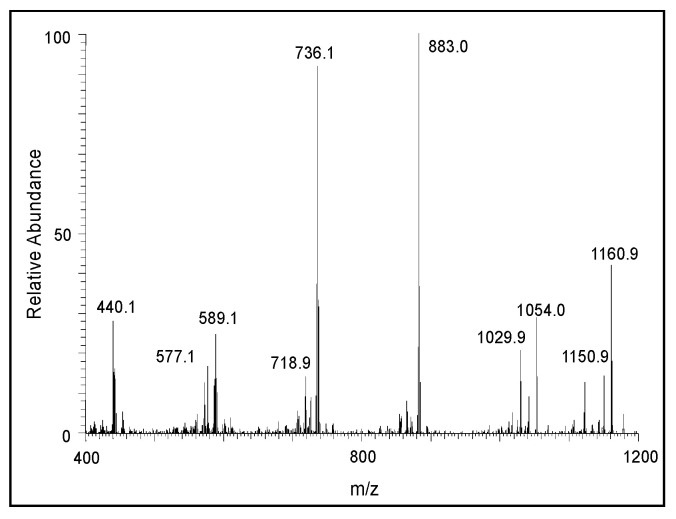
The CID mass spectrum of the ion with an *m/z* value of 1178 amu.

A similar, but much clearer, fragmentation pattern could be observed for the pentameric compound (Figure 9). The fragmentation ions at around 587, 442, and 295 amu correspond to the oxidized form of tetrameric, trimeric, and dimeric DHI units, respectively. The CID of the hexameric product is shown in Figure 10. The product ions at 440, 587, and 734 amu are due to the oxidized form of the trimeric, tetrameric, and pentameric ions, respectively. There is also a prominent decomposition ion at 430 amu, which could be due to the loss of the oxidized form of the trimeric ion and carbon atom (883 − 430 = 453; which is carbon 12 + oxidized trimeric ion with 441). With this information, it is deduced that the parent ion is due to the hexameric compound. The CID of the heptameric compound is shown in Figure 11. The decomposition ions observed at 440, 588, 733, and 881 are due to the oxidized forms of trimeric, tetrameric, pentameric, and hexameric products, respectively. Therefore, the parent ion appears to be a heptameric product. The CID of the last product ion is shown in Figure 12. The prominent ions seen in the CID spectrum are 440, 589, 736, 888, and 1030, which could arise from oxidized forms of the trimeric, tetrameric, pentameric, hexameric, and heptameric compounds, respectively. Thus, this ion is due to an octameric product.

## 3. Discussion

**Figure 13:**
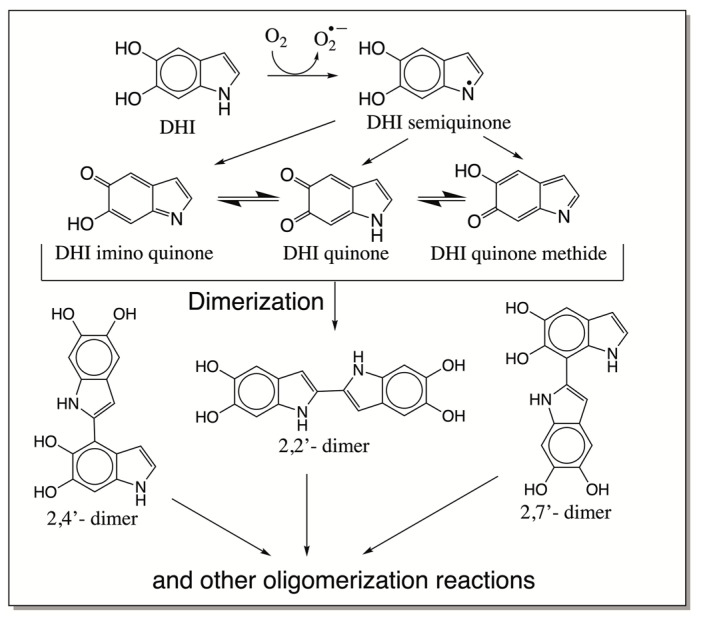
Proposed mechanism for the oxidative polymerization of DHI. DHI undergoes rapid aerial oxidation, producing DHI semiquinone radicals and the superoxide anion. Two molecules of DHI semiquinones undergo a redox reaction, regenerating DHI and producing the two-electron oxidation product that can exist in three different isomeric forms. Rapid condensation of these products generates dimeric, trimeric, and other oligomeric products at initial stages of oxidation. Eventually, they all condense and form insoluble black-colored eumelanin pigment.

From the fragmentation pattern, it is difficult to discern any structural information of the products other than the fact that they are oligomeric products of DHI. In the case of deNADA and related compounds, we were able to establish the benzodioxan-type structure of the dimeric products [36,37,38,39,40]. In these cases, oxidative dimerization and other oligomerizations occur via quinone, quinone methide, and free radical intermediates. In the case of DHI, it is likely that the reaction is proceeded by a free radical mechanism. The extreme sensitivity of DHI to oxygen indicates that the initial reaction is most likely the production of semiquinone radicals and the superoxide anion. Two molecules of semiquinone can easily undergo double decomposition to generate back DHI and produce the two-electron oxidation product, which could exist in three different isomeric forms as shown in Figure 13 [27]. These quinonoid species can form dimeric, trimeric, and other oligomeric products. Accordingly, we were able to witness the production of a number of oligomeric products of DHI.

**Figure 14:**
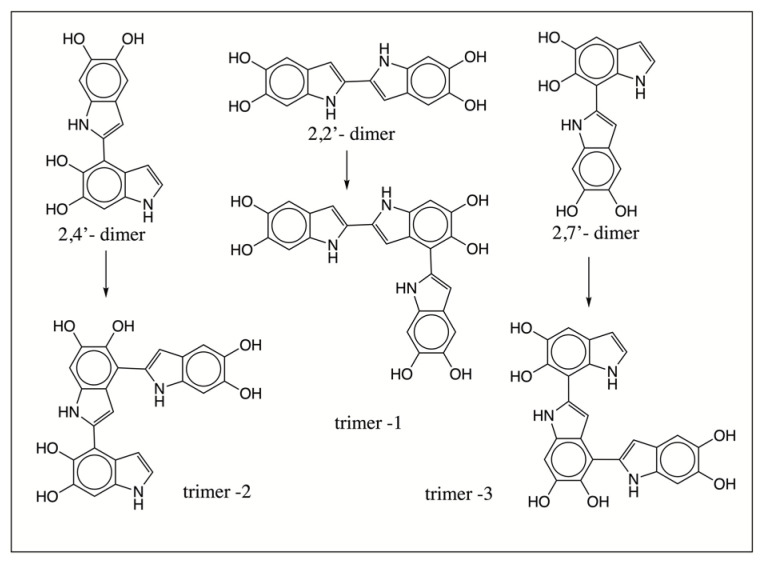
Proposed mechanism for the formation of trimeric products of DHI during nonenzymatic oxidative polymerization of DHI. Different DHI dimeric products generated during the initial stages of oxidation (shown in Figure 13) will undergo further condensation with the oxidized form of DHI, yielding trimeric products of DHI. Only one possible structure of the trimeric product arising from each of the dimeric species is illustrated. However, many other isomeric forms are possible. Interestingly, all of the isomeric compounds will exhibit a similar CID spectrum, losing a DHI unit, as shown in Figure 7.

The mechanism of the oxidative polymerization of DHI has been extensively investigated by Italian scientists using chemical model reactions [26,27,28,41]. They identified that the 2-position of DHI is the most vulnerable to oxidative coupling. Therefore, four possible dimeric products—2,2′-, 2,3′- 2,4′- and 2,7′—linked adducts of DHI can be formed. Of these products, the 2,3′ adduct is least likely to be generated due to electronic considerations (Figure 13). The isolation and characterization of these isomeric adducts proved to be an extremely difficult task. Exhaustive studies carried out on the oxidation of DHI produced only a small amount of 2,4′-dimeric product from the enzymatic reactions for characterization purposes, while isolation and identification of other dimeric compounds proved to be a formidable job. Our current understanding is that both 2,4′- and 2,7′-dimeric compounds are more likely generated during initial stages of polymerization than other products [41]. Since the dimeric products are even more reactive than monomeric DHI, the structural elucidation of trimeric and other oligomeric products remains extremely difficult and complicated. Nevertheless, studies carried out with oxidative dimerization of dimeric compounds indicate the production of tetrameric compounds with more complex structures [41]. Based on current mass spectral data, the course of oxidative polymerization of DHI can be summarized as follows: Nonenzymatic oxidation of DHI will produce different quinonoid products via a semiquinone intermediate. The resultant quinonoid species will undergo facile dimerization to form 2,4′- and 2,7′- dimers (Figure 13). On collision-induced decomposition, they produce an oxidized form of the monomeric ion irrespective of the structural variations (Figure 6). The addition of the monomeric quinonoid form of DHI to the dimeric product will produce a number of trimeric compounds, as shown in Figure 14. Only one of many possible structures of the trimeric product for each of the dimeric product is shown in this figure. They all will fragment with the loss of one DHI unit, producing a major fragmentation ion at 295 amu in their CID spectrum (Figure 7). The process will continue producing more complex oligomeric products.

One interesting observation on the mass spectra of the DHI reaction is the fact that although we were able to see the dimeric and trimeric products of DHI, the subsequent oligomeric compounds were mostly identified as their two electron oxidation products. Thus, the ion observed at *m/*z 589 is due to the oxidized form of the tetrameric product, the ion observed at *m/z* 736 is due to the oxidized form of the pentameric product, and the ion observed at *m/z* 883 is due to the oxidized form of the hexameric product. It is important to stress that higher oligomeric products are in the oxidized form, which will exhibit general absorbance and, thus, accounts for the black coloration of the polymer [27,42]. The ions of subsequent oligomeric products were also observable, but the nature of the redox state of the molecules was difficult to establish. Interestingly, all of the possible oligomeric products up to the undecameric compound were detected (Figure 5), suggesting a stepwise nature of the polymerization, as proposed by Arzillo et al. [43]. Thus, the results presented in this paper indicate the facile oxidation of DHI, even under mild conditions, to dimeric and other oligomeric products. In a biological system, especially in the presence of metal ions, the oxidation could proceed even faster and result in the rapid production of eumelanin pigment [7,42,44]. Occurrence of such reactions will ensure practically instantaneous deposition of eumelanin pigment at the reaction site. This ensures the rapid polymerization of DHI and reduces the possible toxicity of quinonoid intermediates because polymerization reaction diverts the quinonoid compounds from reacting with the cellular nucleophiles.

It is important to draw particular attention to the way insects produce melanin in their system, which is completely different from that of other animals. Mammalian systems use tyrosine and dopa as the major precursors of eumelanin. Insects, on the other hand, use mostly dopamine and not dopa [45]. This difference, coupled with the fact that insect dopachrome decarboxylase/tautomerase makes only DHI [9,14,23,24,25] and not DHICA from dopachrome, dictates the sole production of DHI for eumelanogenesis in insects. Animals also generate melanin in the confinement of melanocytes and transfer melanin to the epidermis for pigmentation. On the other hand, insects and other arthropods do not generate melanin in melanocytes but produce them in the entire open circulatory system of hemolymph. Use for skin pigmentation is only one aspect of melanin biochemistry in insects. Most importantly, melanin is used for two other physiologically important processes, namely wound healing and defense reactions (innate immunity) [10,11,12,13,14,15,16,17]. Massive deposition of melanin at the wound site in cuticle is well documented [13]. This process not only prevents the excessive loss of hemolymph at the wound site but also prohibits the entry of opportunistically invading parasites and other foreign objects [13,16]. Similarly, during infection, foreign organisms are routinely found encapsulated and melanized in the hemolymph of insects as an innate response to infection [11]. The deposition of melanin inhibits the multiplication of foreign bodies and deprives them of any needed nutrients for survival. Moreover, production of reactive quinonoid species from DHI during melanogenesis can have deleterious consequences for the invading organisms. Even though it also poses enormous danger to self-matter, as it could cause depletion of cellular thiols and other cellular nucleophiles, rapid polymerization of DHI will ensure its instantaneous conversion to insoluble melanin pigment necessary to encapsulate and limit the damage caused by intruding parasites and pathogens, while sparing the endogenous materials. Thus, production of DHI seems to be extremely advantageous for insects. Oxidation products of DHICA, on the other hand, will stay in solution for a longer time and may cause damage to the endogenous materials. Therefore, the production of DHI melanin is much more advantageous for insects than the generation of DHICA melanin and/or mixed melanin of DHICA and DHI. Accordingly, insects have managed to avoid the production of DHICA by having specifically dopachrome decarboxylase/tautomerase, which ensures the production of only DHI and no possible DHICA formation in their system (Figure 1) [9,14,23,24,25]. Insects also seem to lack the ability to oxidize DHICA, and hence do not appear to make DHICA melanin [46]. Our recent finding that the majority of melanin pigment formed in insects originates from dopamine (and hence DHI melanin) and not from dopa also supports these conclusions [45]. Thus, the results presented in this paper suggest a key advantage of DHI usage in insects.

## 4. Materials and Methods

HPLC-grade methanol and ammonium formate (99%) were purchased from Acros Organics, Morris Plains, NJ, USA. HPLC-grade water was obtained from a Milli Q synthesis A10 Water purification system purchased from Millipore, Milford, MA, USA. All other chemicals were of analytical grade purchased from Fisher Scientific, Pittsburg, PA, USA and/or VWR International, Radnor, PA, USA. DHI and DHICA were synthesized using potassium ferricyanide oxidation of DL-dopa, as outlined in earlier publications [1,47]. A solution of K_3_[Fe(CN)_6_], and NaHCO_3_ in water was poured into a solution of DL-dopa, (all samples had been separately purged with an argon flux for 10 min) while stirring at room temperature. For the preparation of DHI, the wine-red solution of dopachrome was stirred at room temperature for 2 h. Solid Na_2_S_2_O_5_ was then added to the dark-brown solution, which was extracted with ethyl acetate. Evaporation of ethyl acetate extracts produced a brown oil, which was dissolved in acetone plus benzene; hexane was added to the solution. After the filtration of black melanin precipitate, the resulting brown solution was gradually mixed with hexane to produce almost colorless crystals of DHI (61% yield). For the preparation of DHICA, the wine-red solution of dopachrome was stirred for 2 min and mixed with 1 M NaOH, which had been degassed by purging with an argon flux for 10 min. After 15 min at room temperature, solid Na_2_S_2_O_5_ and then 6 M HCl were added to the dark brown solution. The resulting pale brown solution was extracted with ethyl acetate. Evaporation of ethyl acetate produced a purple crystalline powder, which was dissolved in acetone, and hexane was added. After the filtration of dark-brown melanin precipitate, the resulting brown solution was gradually mixed with hexane to produce almost colorless crystals of DHICA (74% yield).

Reaction Conditions: A reaction mixture containing 100 nmol of DHI in 1 mL of water at room temperature was incubated for 10 min, and an aliquot of the reaction (100 μL) was quenched with (900 μL) 1% trifluoroacetic acid. This diluted mixture was subjected to electrospray tandem mass spectrometric (ESI-MS/MS) analysis. The diluted reaction was directly injected into the mass spectrometer. A Thermo Finnigan LCQ Advantage electrospray ion trap mass spectrometer (Sunnyvale, CA, USA) was used to detect and characterize the products. The operating conditions of the ion trap mass spectrometer are: capillary temperature of 280 °C; a spray voltage of 4.00 kV; and sheath gas of 30 cm^3^/min. Collision-induced dissociation (CID) was performed at a relative collision energy of 28, an isolation mass window of 2.5 amu, and a default activation Q and activation time of 0.25 and 30 ms, respectively.

## Abbreviations

CID	Collision-induced decomposition
deNADA	1,2-dehydro-*N*-acetyldopamine
DHI	5,6-dihydroxyindole
DHICA	5,6-dihydroxyindole-2-carboxylic acid
